# Robot-Assisted Minimally Invasive Esophagectomy (RAMIE) vs. Conventional Minimally Invasive Esophagectomy (MIE) for Esophageal Cancer: A Nationwide Inpatient Sample Analysis from 2017 to 2020

**DOI:** 10.5761/atcs.oa.25-00017

**Published:** 2025-05-23

**Authors:** Weizhong Ruan, Yibin Cai, Weisheng Chen

**Affiliations:** Department of Thoracic Surgery, Fujian Cancer Hospital, Clinical Oncology School of Fujian Medical University, Fuzhou, Fujian, China

**Keywords:** robot-assisted, minimally invasive esophagectomy (MIE), esophageal cancer, nationwide inpatient sample (NIS)

## Abstract

**Purpose:** This study compared the short-term outcomes after conventional minimally invasive esophagectomy (MIE) vs. robot-assisted minimally invasive esophagectomy (RAMIE)s by analyzing national data.

**Methods:** Data were collected from adults aged ≥20 years who underwent MIE from 2017 to 2020, from the US Nationwide Inpatient Sample database. The outcomes included in-hospital mortality, unfavorable discharges, prolonged length of stays (LOS), total hospital charge, and various complications. Propensity score matching (PSM) was employed to balance the baseline characteristics between RAMIE and conventional MIE.

**Results:** After PSM, 628 patients (representing 3140 patients in the US after weighting) were analyzed. After adjustment, multivariable analysis revealed no significant differences between RAMIE and traditional MIE in terms of in-hospital mortality (adjusted odd ratio [aOR] =1.45, 95% confidence interval [CI]: 0.46–4.61), unfavorable discharge (aOR = 0.76, 95%CI: 0.41–1.41), prolonged LOS (aOR = 0.87, 95%CI: 0.60–1.26), total hospital charge (aBeta = 12.23, 95%CI: −19.24 to 43.69), or complications (aOR = 1.05, 95%CI: 0.78–1.41). Stratified analysis indicated that, among obese patients, RAMIE was associated significantly with a higher risk of overall complications compared with MIE (aOR = 1.90, 95%CI: 1.11–3.25).

**Conclusions:** The study found no significant differences in unfavorable discharge and prolonged LOS between RAMIE and traditional MIE. Nevertheless, obese patients undergoing RAMIE experienced higher complications.

## Introduction

Esophageal cancer is the sixth leading cause of cancer-related deaths worldwide, with an incidence rate of 6.3 per 100000.^1,2)^ It is often diagnosed at an advanced stage, thus entailing a poor prognosis and high mortality rates.^3)^ Risk factors, such as obesity, alcohol, and tobacco, contribute to the initiation of adenocarcinoma in the lower two thirds of the esophagus, whereas smoking, certain dietary factors/habits, and genetic factors raise the risk of squamous cell carcinoma in the upper esophagus, while poor nutritional status places individuals at elevated risk for either category of esophageal malignancy.^1,2,4)^ The mainstay of curative treatment for esophageal cancer includes surgical resection,^5)^ which historically has been performed via open surgery, leading to substantial morbidity and prolonged recovery.^6)^

Minimally invasive esophagectomy (MIE) has emerged as a favorable alternative, as it offers reduced postoperative pain, shorter hospital stays, and fewer respiratory complications compared with traditional open esophagectomy.^7,8)^ Performed laparoscopically, MIE has demonstrated oncological outcomes comparable with those of open surgery.^9)^ The evolution of MIE has been marked by the introduction of robotic systems, which aim to enhance the precision of minimally invasive techniques.^10)^ Moreover, robot-assisted minimally invasive esophagectomy (RAMIE) integrates advanced robotic technology potentially to improve the visual field, while increasing dexterity, and stabilizing instrument movement, which may facilitate complex dissections and suturing during esophagectomy.^11,12)^

While MIE has demonstrated promising results compared with open surgery, the role of RAMIE in enhancing these benefits is an area of ongoing investigation. Using the dataset from a large nationally representative database, this study aimed to compare the in-hospital outcomes of RAMIE and conventional MIE for esophageal cancer. These outcomes are crucial markers of surgical success and patient recovery and could provide valuable insights into the comparative effectiveness and safety profiles of these two minimally invasive approaches.

## Materials and Methods

### Data source

The nationwide inpatient sample (NIS) is part of the Healthcare Cost and Utilization Project (HCUP), sponsored by the Agency for Healthcare Research and Quality (AHRQ). This comprehensive database is the largest publicly available all-payer inpatient healthcare database in the United States (US). It is designed to produce regional and national estimates of inpatient utilization, access, charges, quality, and outcomes. The NIS database captures information from approximately 7 million hospital stays each year and encompasses more than 1000 hospitals, sampled to approximate a 20% stratified sample of US community hospitals. This sampling frame allows the system to estimate over 36 million hospitalizations nationally when data are weighted, thus reflecting a broad spectrum of hospital settings, regions, and populations.

### Study design and population

We conducted a sampling-based, retrospective cohort study using data extracted from the NIS database. The study included hospitalized adults aged 20 years or older who underwent MIE between 2017 and 2020. Patients were excluded if they were admitted on an emergent basis, diagnosed with metastatic cancer (including metastasis to lymph nodes), or had incomplete records of age, sex, and mortality outcomes. All diagnoses and procedures were identified using the International Classification of Diseases, ninth revision (ICD-9), and tenth revision (ICD-10), as detailed in **Supplemental Table S1**.

### Ethics statement

The study utilized de-identified patient data accessed through a formal request to the Online HCUP Central Distributor (website: https://www.distributor.hcup-us.ahrq.gov/, certificate # HCUP-328J53GZS). The use of data adhered strictly to the terms of the data-use agreement specified by the NIS. The study was conducted without direct patient or public involvement and focused on secondary data analysis of the NIS database. Our hospital's Institutional Review Board reviewed the study protocol and granted an exemption from further review and informed consent, because anonymized data were utilized.

### Variables and outcome measures

The study focused on outcomes including in-hospital mortality, unfavorable discharge (i.e., discharged to long-term care facilities), prolonged length of stay (LOS) (defined as ≥75th LOS in the study sample), total hospital charge, and complication rates. Complications consisted of anastomotic leak, arrhythmia, infection, sepsis, pneumonia, respiratory failure, mechanical ventilation, dysphagia, venous thromboembolism, cerebrovascular accident, acute myocardial infarction, bleeding, shock, urinary tract infection, and acute kidney injury.

Demographic and hospital-related variables were collected, including patient age, sex, insurance status, smoking status, hospital bed numbers (detailed definition is documented in **Supplemental Table S2**), location/teaching status, and region. The analysis was stratified by age (younger than 60 years and 60 years or older) and obesity status to explore differences in outcomes among these subgroups.

### Statistical analysis

Descriptive statistics of the patients’ demographical and clinical characteristics are presented in unweighted counts (n) and weighted percentage (%) or mean ± standard error. Categorical variables were compared using the Rao–Scott chi-square test, while continuous variables were analyzed using survey methods that incorporate stratification, clustering, and sampling weights, ensuring valid and robust statistical inferences for complex survey designs. A propensity score matching (PSM) at a 1:1 ratio was computed based on the probability of RAMIE, adjusting for variables with a significance level of *p* <0.05 as detailed in **Supplementary Table S1**. The matching process employed a one-to-many approach, prioritizing the “best” matches first and subsequently proceeding with the “next-best” matches until no further matches were possible. Logistic regression analysis was used to calculate odds ratios (ORs) and 95% confidence intervals (CIs) for binary outcomes. Linear regression analysis was conducted to assess the association between study variables and total hospital charges. Multivariable analyses adjusted for variables that still showed between-group significance after PSM (*p* <0.05). Stratified analysis was employed to identify the association between surgical types and outcomes across the patient subgroups of different ages and obesity status. All *p* values were two-sided and *p* < 0.05 was considered statistically significant. All statistical analyses were performed using the statistical software package SAS software version 9.4 (SAS Institute Inc., Cary, NC, USA).

## Results

### Study population selection

**Fig. 1:**
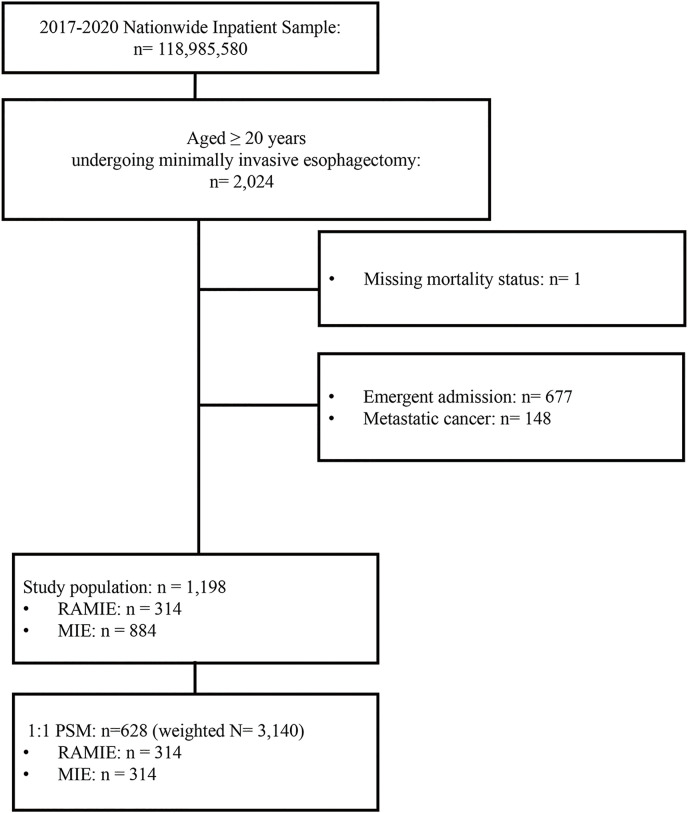
Flow diagram of study cohort selection. A total of 2024 hospitalized patients aged ≥20 years who underwent minimally invasive esophagectomy from the NIS from 2017 to 2020 were included. Patients with missing information on study outcomes of interest (n = 1) were excluded. We further excluded 677 patients who had an emergent admission and 148 patients with metastatic cancers. Finally, 1198 patients were included. Of these, 314 (26.2%) patients underwent RAMIE. After 1:1 PSM, 628 patients remained and were included in the analysis. This study sample represents a total of 3140 hospitalized patients in the entire US after applying the weighting method guided by the NIS database. NIS: nationwide inpatient sample; PSM: propensity score matching; RAMIE: robot-assisted minimally invasive esophagectomy

The study population selection process is depicted in **Fig. 1**. A total of 2024 hospitalized patients aged ≥20 years who underwent MIE from the NIS from 2017 to 2020 were included. Patients with missing information on study outcomes of interest (n = 1) were excluded. We further excluded 677 patients who had an emergent admission and 148 patients with metastatic cancers. Finally, 1198 patients were included. Of these, 314 (26.2%) patients underwent RAMIE. **Supplemental Table S3** showed comparisons of study population before PSM. After 1:1 PSM according to age (in years), hypothyroidism, and hospital bed numbers, 628 patients remained and were included in the analysis. This study sample represents a total of 3140 hospitalized patients in the entire US after applying the weighting method guided by the NIS database (**Fig. 1**).

### Patient characteristics

In-hospital outcomes, demographics, hospital-related information, and major comorbidities are summarized in **Table 1** and **Supplementary Table S3**. Before PSM, the mean age of all patients was 63.4 years, 62.7% of patients were male, and 47.2% of patients had at least one comorbidity (**Supplementary Table S3**). After PSM, the study variables were balanced, where hypertension remained significantly different between the two groups (**Table 1**).

**Table 1:** Characteristics of the study population after propensity score matching

Characteristics	Total (*n* = 628)	Surgical types		*p*-value
		RAMIE (*n* = 314)	MIE (*n* = 314)	
Outcomes				
In-hospital mortality	10 (1.6)	6 (1.9)	4 (1.3)	0.469
Unfavorable discharge ^a^	38 (6.1)	17 (5.5)	21 (6.8)	0.480
Prolonged LOS ^a,b^	134 (21.7)	64 (20.8)	70 (22.6)	0.573
Total hospital charge (1000 USD)	179.7 ± 7.8	187.9 ± 7.7	171.5 ± 9.2	0.266
Complications	302 (48.1)	152 (48.4)	150 (47.8)	0.866
Anastomotic leak	36 (5.7)	18 (5.7)	18 (5.7)	1.000
Arrhythmia	83 (13.2)	44 (14.0)	39 (12.4)	0.547
Infection	63 (10.0)	27 (8.6)	36 (11.5)	0.179
Sepsis	26 (4.1)	12 (3.8)	14 (4.5)	0.653
Pneumonia	28 (4.5)	14 (4.5)	14 (4.5)	1.000
Respiratory failure	59 (9.4)	33 (10.5)	26 (8.3)	0.293
Mechanical ventilation	41 (6.5)	25 (8.0)	16 (5.1)	0.091
Dysphagia	123 (19.6)	70 (22.3)	53 (16.9)	0.076
VTE	20 (3.2)	10 (3.2)	10 (3.2)	1.000
CVA	2 (0.3)	2 (0.6)	0 (0.0)	–
AMI	3 (0.5)	0 (0.0)	3 (1.0)	–
Bleeding	3 (0.5)	0 (0.0)	3 (1.0)	–
Shock	20 (3.2)	10 (3.2)	10 (3.2)	1.000
Urinary tract infection	14 (2.2)	5 (1.6)	9 (2.9)	0.251
Acute kidney injury	46 (7.3)	21 (6.7)	25 (8.0)	0.514
Demography				
Age, years	61.7 ± 0.5	61.5 ± 0.6	61.9 ± 0.7	0.751
20–29	9 (1.4)	6 (1.9)	3 (1.0)	0.817
30–39	32 (5.1)	13 (4.1)	19 (6.1)	
40–49	63 (10.0)	33 (10.5)	30 (9.6)	
50–59	141 (22.5)	72 (22.9)	69 (22.0)	
60–69	177 (28.2)	89 (28.3)	88 (28.0)	
70–79	177 (28.2)	87 (27.7)	90 (28.7)	
80+	29 (4.6)	14 (4.5)	15 (4.8)	
Sex				0.605
Male	416 (66.2)	211 (67.2)	205 (65.3)	
Female	212 (33.8)	103 (32.8)	109 (34.7)	
Insurance status / Primary payer				0.587
Medicare/Medicaid	331 (52.9)	170 (54.5)	161 (51.3)	
Private including HMO	270 (43.1)	129 (41.3)	141 (44.9)	
Self-pay/no-charge/other	25 (4.0)	13 (4.2)	12 (3.8)	
Missing	2	2	0	
Smoking	319 (50.8)	164 (52.2)	155 (49.4)	0.446
Major comorbidities				
Congestive heart failure	33 (5.3)	17 (5.4)	16 (5.1)	0.834
Hypertension	350 (55.7)	186 (59.2)	164 (52.2)	**0.044**
Hypothyroidism	39 (6.2)	23 (7.3)	16 (5.1)	0.225
Diabetes	125 (19.9)	66 (21.0)	59 (18.8)	0.444
Obesity	142 (22.6)	70 (22.3)	72 (22.9)	0.836
Chronic pulmonary disease	129 (20.5)	60 (19.1)	69 (22.0)	0.320
Chronic kidney disease	41 (6.5)	25 (8.0)	16 (5.1)	0.127
Severe liver disease	1 (0.2)	1 (0.3)	0 (0.0)	–
Rheumatic disease	16 (2.5)	9 (2.9)	7 (2.2)	0.584
Weekend admission	6 (1.0)	2 (0.6)	4 (1.3)	0.282
Hospital bed numbers				0.781
Small	81 (12.9)	43 (13.7)	38 (12.1)	
Medium	73 (11.6)	36 (11.5)	37 (11.8)	
Large	474 (75.5)	235 (74.8)	239 (76.1)	
Location/teaching status				0.643
Rural	12 (1.9)	7 (2.2)	5 (1.6)	
Urban nonteaching	45 (7.2)	21 (6.7)	24 (7.6)	
Urban teaching	571 (90.9)	286 (91.1)	285 (90.8)	

Continuous variables are presented as mean ± SE.

Categorical variables are presented as unweighted counts (weighted percentages).

*P*-values <0.05 are shown in bold.

^a^ Excluded in-hospital mortality patients.

^b^ Length of stay >75th percentile (10 days).

RAMIE: robot-assisted minimally invasive esophagectomy; MIE: conventional minimally invasive esophagectomy; LOS: length of stay; VTE: venous thromboembolism; CVA: cerebrovascular accident; AMI: acute myocardial infarction

### Associations between surgical types and in-hospital outcomes

Associations between surgical types and outcomes in patients are summarized in **Table 2**. After adjusting for relevant confounders in the multivariable analysis, RAMIE was not significantly associated with the risks of unfavorable discharge (adjusted odd ratio [aOR] = 0.76, 95% CI: 0.41–1.41, *p* = 0.389) and prolonged LOS (aOR = 0.87, 95% CI: 0.60–1.26, *p* = 0.452) as compared to MIE (**Table 2**).

**Table 2:** Associations between surgical types (RAMIE vs. MIE) and in-hospital outcomes

Outcomes	aOR/aBeta ^c,d^ (95% CI)	*p*-value
In-hospital mortality	1.45 (0.46–4.61)	0.526
Unfavorable discharge ^a^	0.76 (0.41–1.41)	0.389
Prolonged LOS ^a,b^	0.87 (0.60–1.26)	0.452
Total hospital charge (1000 USD)	12.23 (−19.24–43.69)	0.446
Complications, any	0.99 (0.73–1.34)	0.937
Anastomotic leak	0.96 (0.51–1.81)	0.897
Arrhythmia	1.10 (0.70–1.75)	0.676
Infection	0.70 (0.44–1.13)	0.140
Sepsis	0.81 (0.40–1.65)	0.556
Pneumonia	1.01 (0.52–1.97)	0.985
Respiratory failure	1.29 (0.79–2.10)	0.310
Mechanical ventilation	1.51 (0.86–2.63)	0.149
Dysphagia	1.41 (0.96–2.08)	0.078
VTE	0.97 (0.42–2.28)	0.950
Shock	0.93 (0.45–1.92)	0.845
Urinary tract infection	0.55 (0.19–1.58)	0.262
Acute kidney injury	0.78 (0.43–1.40)	0.393

^a^ Excluded patients with in-hospital mortality.

^b^ Length of stay >75th percentile (10 days).

^c^ Beta was used to total hospital charge.

^d^ Adjusted for related variables of *p*-value <0.05 in **Table 1**, including hypertension.

RAMIE: robot-assisted minimally invasive esophagectomy; MIE: conventional minimally invasive esophagectomy; LOS: length of stay; VTE: venous thromboembolism

### Association between surgical types and in-hospital outcomes, stratified by obesity and age

We further carried out a stratified analysis by obesity status and age (<60 years and ≥60 years). The results are shown in **Table 3**. After adjusting for confounders, obese patients who underwent RAMIE had a significantly higher risk for complications (aOR = 1.75, 95% CI: 1.08–2.86, *p* = 0.025) compared to those who underwent MIE. No significant difference was observed in in-hospital outcomes between the two surgical types among patients both aged <60 years and ≥60 years (**Table 3**).

**Table 3:** Stratified association between surgical types (RAMIE vs. MIE) and in-hospital outcomes by obesity status and age

Outcomes	aOR/aBeta ^c^ (95% CI)	*p*-value
Without obesity		
In-hospital mortality	1.92 (0.52–7.17)	0.329
Unfavorable discharge ^a^	0.67 (0.34–1.30)	0.234
Prolonged LOS ^a,b^	0.94 (0.61–1.43)	0.763
Total hospital charge	24.33 (−10.11–58.78)	0.166
Complications, any	0.83 (0.60–1.15)	0.261
With obesity		
In-hospital mortality	NA	–
Unfavorable discharge ^a^	NA	–
Prolonged LOS ^a,b^	0.61 (0.33–1.13)	0.114
Total hospital charge	−29.40 (−101.98–43.17)	0.425
Complications, any	**1.75 (1.08**–**2.86)**	**0.025**
Aged <60 y		
In-hospital mortality	0.48 (0.04–5.81)	0.560
Unfavorable discharge ^a^	1.44 (0.42–4.97)	0.561
Prolonged LOS ^a,b^	1.20 (0.69–2.08)	0.510
Total hospital charge	5.78 (−40.33–58.89)	0.805
Complications, any	1.31 (0.86–1.99)	0.206
Aged ≥60 y		
In-hospital mortality	2.54 (0.55–11.68)	0.230
Unfavorable discharge ^a^	0.71 (0.36–1.40)	0.320
Prolonged LOS ^a,b^	0.75 (0.48–1.16)	0.195
Total hospital charge	16.54 (−25.82–58.89)	0.443
Complications, any	0.84 (0.58–1.20)	0.336

*P*-values <0.05 are shown in bold.

^a^ Excluded patients with in-hospital mortality.

^b^ Length of stay >75th percentile (10 days).

^c^ Beta was used to total hospital charge.

^d^ Adjusted for related variables of *p*-value <0.05 in **Table 1**, including hypertension.

NA: no event occurred in one group; RAMIE: robot-assisted minimally invasive esophagectomy; MIE: conventional minimally invasive esophagectomy; LOS: length of stay

## Discussion

This sampling-based, large scale study provides comprehensive insights into the comparative effectiveness and potential benefits of RAMIE vs. conventional MIE for esophageal cancer. The current study is of special significance, as there have been few large-scale randomized control trials comparing RAMIE and conventional MIE. The findings suggest that RAMIE and MIE are comparable in terms of in-hospital mortality, discharge outcomes, LOS, and hospital charge, with no significant differences detected between the two surgical techniques. However, further subgroup analyses revealed that, among obese patients, specifically, RAMIE appears to be associated with slightly increased complication rates than MIE. These findings suggest the need for careful consideration when selecting the surgical technique for obese individuals with esophageal cancer, potentially benefiting personalized treatment decisions.

Echoing our findings, a 2020 analysis encompassing 100 patients reported no statistically significant discrepancies in postoperative complications when comparing RAMIE and MIE for esophageal cancer surgery.^13)^ A larger retrospective study by Lei et al. involving 270 patients with esophageal squamous cell carcinoma reinforced this observation, yet revealed that RAMIE may be more advantageous than MIE in achieving more extensive lymph node dissection—particularly regarding the number of thoracic nodes retrieved^14)^ Nevertheless, both studies were constrained by relatively modest sample sizes and single-center designs, underscoring the need for more expansive, multi-institutional research to validate these findings.

In a recent review, Chin et al. observed that RAMIE may confer improved postoperative outcomes, including reduced length of hospital stay and more favorable cosmetic results.^15)^ Another newly published review similarly suggested that overall complication rates—encompassing dysphagia, anastomotic leakage, infection, thrombosis, hemorrhage, and pneumonia,^16)^ as well as chyle leak, recurrent laryngeal nerve palsy, delayed gastric emptying, diaphragmatic herniation, and stricturing^17)^—are at least comparable, and potentially lower, with RAMIE compared to open esophagectomy or conventional thoracoscopic MIE.^18)^ Notably, a recent propensity score–matched analysis reported no statistically significant difference in the incidence of major complications (namely anastomotic leakage) between matched and unmatched cohorts.^19)^

A 2021 analysis highlighted a markedly lower rate of pulmonary complications in patients undergoing RAMIE (18%) compared with conventional MIE (44%), while also demonstrating a substantially decreased incidence of recurrent laryngeal nerve palsy in the RAMIE cohort (7% vs. 20% for conventional MIE).^20)^ Another RAMIE trial evaluating Chinese patients with esophageal squamous cell carcinoma, comparable incidences of pulmonary complications, anastomotic leakage, and recurrent laryngeal nerve paralysis were observed in both RAMIE and MIE. Based on these findings, the investigators concluded that both approaches are safe and feasible treatment strategies for this patient population.^21)^

Notably, our stratified analysis suggested an elevated complication risk for RAMIE compared with MIE among obese patients, likely reflecting the specific technical challenges posed by obesity in robotic surgery—namely restricted visualization and difficulties in port placement.^22)^ Although additional investigations are necessary to validate this hypothesis, obesity may impede the precise maneuverability of robotic instruments, potentially prolonging operative times and amplifying complication rates. Excess adipose tissue can also obscure anatomical landmarks, thus reducing visibility and precision while heightening the risk of inadvertent injury. Furthermore, the placement of robotic ports may be compromised in obese patients, leading to suboptimal positioning and increased tension on the instruments. Finally, anesthesia management is often more complex in this population, owing to difficulties in airway management and a higher prevalence of comorbid conditions.^23)^

Robotic systems are generally recognized as more expensive due to the cost of the robot itself and its maintenance. However, our analysis did not show higher total hospital charge of RAMIE group. This result might be influenced by how hospitals handle financials, such as amortizing costs over many procedures or bundling charges differently, which can obscure direct cost comparisons. Additionally, hospital reimbursement structures, particularly if they cap charges for certain procedures, might mask the typically higher costs associated with robotic surgeries. The current analysis cannot identify specific cost factors due to the lack of granular data in the dataset. Further analysis, such as cost-effectiveness studies, may be necessary to better understand the financial impacts of these surgical methods.

### Strengths and limitations

The present study utilized a large, nationally representative database, which enhances the generalizability of the findings to the US adult population undergoing MIE. Additionally, the application of PSM helps minimize biases by balancing the cohorts based on various relevant variables. This approach ensures a more accurate comparison between the different surgical modalities. Additionally, the large sample size enabled us to evaluate a broad spectrum of complications, from more common issues such as dysphagia to less frequent but clinically significant ones like arrhythmias and anastomotic leakage, thereby enhancing the value of this study in the existing literature. Our stratified analysis on obesity, which has not been previously evaluated in this context within the medical literature, further highlights that selecting between the two procedures should be tailored to patients’ characteristics to optimize outcomes.

However, the study does entail several limitations that need to be considered. First, its reliance on administrative data can lead to potential inaccuracies, due to coding errors or inconsistencies with respect to how data are recorded between different hospitals. This issue might affect the precision of identifying specific patient conditions or procedural details. Furthermore, the retrospective design of the study could introduce selection bias and there is an issue of potential residual confounding, as not all relevant variables may be captured measured accurately in the administrative database. Some crucial intraoperative parameters such as lymph node clearance, operation time, or exact blood loss volume, could not be captured. The use of data from a single country may limit the global applicability of the findings, as healthcare systems and practices vary internationally. Another limitation of this study is the inability to distinguish between different esophagectomy techniques (e.g., Ivor Lewis vs. McKeown), as this level of detail is not available in the NIS database. Given that these approaches differ in terms of anastomotic site and potential complication profiles, future studies with more detailed procedural classifications are warranted to further elucidate their impact on patient outcomes. The analysis was also limited by the absence of tumor staging and histological subtype data, as well as information on radiation and neoadjuvant chemoradiotherapy in the NIS database. Given that these factors influence in-hospital outcomes, the findings should be interpreted with caution. Finally, it should be noted that the analysis was limited to in-hospital outcomes, which do not capture long-term oncological outcomes, survival, or quality of life post-discharge.

## Conclusion

In the US, RAMIE and conventional MIE for treating esophageal cancer are comparable in terms of in-hospital mortality, discharge outcomes, LOS, and hospital charge. However, RAMIE is associated with a higher complication rate among obese patients. Future prospective research that includes evaluation of long-term outcomes is warranted to fully assess the benefits and risks of these surgical approaches. There is also a need for more nuanced cost-effectiveness studies to understand the financial and clinical trade-offs of these technologies.

## Declarations

### Ethics approval and consent to participate

The study utilized de-identified patient data accessed through a formal request to the Online HCUP Central Distributor (website: https://www.distributor.hcup-us.ahrq.gov/, certificate # HCUP-328J53GZS). The use of data adhered strictly to the terms of the data-use agreement specified by the NIS. The study was conducted without direct patient or public involvement and focused on secondary data analysis of the NIS database. Our hospitalʼs Institutional Review Board reviewed the study protocol and granted an exemption from further review and informed consent, because anonymized data were utilized.

### Consent for publication

Not applicable.

### Data availability

The datasets analyzed during the current study are available from the corresponding author on reasonable request.

### Conflicts of interest

The authors declare that they have no competing interests.

### Funding

None.

### Author contributions

Conception and design: all authors, Acquisition of data: all authors, Analysis and interpretation of data: all authors, Drafting of the manuscript: all authors, Critical revision of the manuscript: all authors, Final approval of the manuscript: all authors, Guarantor of integrity of the entire study: Weizhong Ruan, Statistical analysis: all authors, Literature research: all authors, Administrative, technical or material support: all authors.

## Supplementary Material

Supplemental Table S1. ICD codes used to define the diagnoses and procedures in the study.

Supplemental Table S2. Categories of hospital bed numbers

Supplemental Table S3. Characteristics of the study population before propensity score matching.

## References

[1] Morgan E Soerjomataram I Rumgay H The global landscape of esophageal squamous cell carcinoma and esophageal adenocarcinoma incidence and mortality in 2020 and projections to 2040: new estimates from GLOBOCAN 2020. Gastroenterology 2022; 163: 649–658.e2. 35671803 10.1053/j.gastro.2022.05.054

[2] Uhlenhopp DJ Then EO Sunkara T Epidemiology of esophageal cancer: update in global trends, etiology and risk factors. Clin J Gastroenterol 2020; 13: 1010–21. 32965635 10.1007/s12328-020-01237-x

[3] Farrow NE Raman V Jawitz OK Impact of age on surgical outcomes for locally advanced esophageal cancer. Ann Thorac Surg 2021; 111: 996–1003. 32853569 10.1016/j.athoracsur.2020.06.055 PMC8023276

[4] Lander S Lander E Gibson MK. Esophageal cancer: overview, risk factors, and reasons for the rise. Curr Gastroenterol Rep 2023; 25: 275–9. 37812328 10.1007/s11894-023-00899-0

[5] Sihag S. Advances in the surgical management of esophageal cancer. Hematol Oncol Clin North Am 2024; 38: 559–68. 38582720 10.1016/j.hoc.2024.03.001

[6] Borggreve AS Kingma BF Domrachev SA Surgical treatment of esophageal cancer in the era of multimodality management. Ann N Y Acad Sci 2018; 1434: 192–209. 29761863 10.1111/nyas.13677

[7] Thomas PA. Milestones in the history of esophagectomy: from torek to minimally invasive approaches. Medicina (Kaunas) 2023; 59: 1786. 37893504 10.3390/medicina59101786 PMC10608184

[8] Yip HC Shirakawa Y Cheng CY Recent advances in minimally invasive esophagectomy for squamous esophageal cancer. Ann N Y Acad Sci 2020; 1482: 113–20. 32783237 10.1111/nyas.14461

[9] Till BM Grenda TR Okusanya OT Robotic minimally invasive esophagectomy. Thorac Surg Clin 2023; 33: 81–8. 36372536 10.1016/j.thorsurg.2022.09.004

[10] Bograd AJ Molena D. Minimally invasive esophagectomy. Curr Probl Surg 2021; 58: 100984. 34629156 10.1016/j.cpsurg.2021.100984 PMC9089813

[11] van Boxel GI Kingma BF Voskens FJ Robotic-assisted minimally invasive esophagectomy: past, present and future. J Thorac Dis 2020; 12: 54–62. 32190354 10.21037/jtd.2019.06.75 PMC7061186

[12] Zhang Y Dong D Cao Y Robotic versus conventional minimally invasive esophagectomy for esophageal cancer: a meta-analysis. Ann Surg 2023; 278: 39–50. 36538615 10.1097/SLA.0000000000005782

[13] Tagkalos E Goense L Hoppe-Lotichius M Robot-assisted minimally invasive esophagectomy (RAMIE) compared to conventional minimally invasive esophagectomy (MIE) for esophageal cancer: a propensity-matched analysis. Dis Esophagus 2020; 33: doz060. 31206577 10.1093/dote/doz060

[14] Lei J Bai Y Qiao Z Robot-assisted minimally invasive esophagectomy versus minimally invasive esophagectomy for thoracic lymph node dissection in patients with squamous cell carcinoma: a retrospective comparative cohort study. J Thorac Dis 2024; 16: 2115–24. 38617764 10.21037/jtd-24-201 PMC11009590

[15] Chin D Shreya S Rusidanmu A Comparing surgical outcomes between conventional thoracoscopic esophagectomy vs. robotic esophagectomy: a review. Clin Oncol 2024; 9: 2060.

[16] Hosoda K Niihara M Harada H Robot-assisted minimally invasive esophagectomy for esophageal cancer: meticulous surgery minimizing postoperative complications. Ann Gastroenterol Surg 2020; 4: 608–17. 33319150 10.1002/ags3.12390 PMC7726681

[17] van Boxel G van Hillegersberg R Ruurda J. Outcomes and complications after robot-assisted minimally invasive esophagectomy. J Vis Surg 2019; 5: 21.

[18] Watanabe M Kuriyama K Terayama M Robotic-assisted esophagectomy: current situation and future perspectives. Ann Thorac Cardiovasc Surg 2023; 29: 168–76. 37225478 10.5761/atcs.ra.23-00064 PMC10466119

[19] Sakurai T Hoshino A Miyoshi K Long-term outcomes of robot-assisted versus minimally invasive esophagectomy in patients with thoracic esophageal cancer: a propensity score-matched study. World J Surg Oncol 2024; 22: 80. 38504312 10.1186/s12957-024-03358-w PMC10953063

[20] Tsunoda S Obama K Hisamori S Lower incidence of postoperative pulmonary complications following robot-assisted minimally invasive esophagectomy for esophageal cancer: propensity score-matched comparison to conventional minimally invasive esophagectomy. Ann Surg Oncol 2021; 28: 639–47. 32892268 10.1245/s10434-020-09081-6

[21] Yang Y Li B Yi J Robot-assisted versus conventional minimally invasive esophagectomy for resectable esophageal squamous cell carcinoma: early results of a multicenter randomized controlled trial: the RAMIE trial. Ann Surg 2022; 275: 646–53. 34171870 10.1097/SLA.0000000000005023

[22] Cofran L Cohen T Alfred M Barriers to safety and efficiency in robotic surgery docking. Surg Endosc 2022; 36: 206–15. 33469695 10.1007/s00464-020-08258-0 PMC8286975

[23] Seyni-Boureima R Zhang Z Antoine M A review on the anesthetic management of obese patients undergoing surgery. BMC Anesthesiol 2022; 22: 98. 35382771 10.1186/s12871-022-01579-8 PMC8985303

