# Supplementary material for: Robot-Assisted Minimally Invasive Esophagectomy (RAMIE) vs. Conventional Minimally Invasive Esophagectomy (MIE) for Esophageal Cancer: A Nationwide Inpatient Sample Analysis from 2017 to 2020
Source: Ann Thorac Cardiovasc Surg. 2025 May 23;31(1):25-00017. doi: 10.5761/atcs.oa.25-00017 (PMC12104546; doi:10.5761/atcs.oa.25-00017)
Supplement: Supplemental Table S1. [file atcs-31-1-25-00017-s01.pdf]

Supplemental Table S1. ICD codes used to define the diagnoses and procedures in the study.

Diagnosis/procedure	ICD-10-CM / ICD-10-PCS
MIE	ICD-10-PCS: 0DB54ZZ, 0DB14ZZ, 0DB24ZZ, 0DB34ZZ, 0DB58ZZ, 0DB18ZZ, 0DB28ZZ, 0DB38ZZ
RAMIE	ICD-10-PCS: (0DB54ZZ, 0DB14ZZ, 0DB24ZZ, 0DB34ZZ, 0DB58ZZ, 0DB18ZZ, 0DB28ZZ, 0DB38ZZ) **AND** (8E0WXCZ, 8E0W3CZ, 8E0W4CZ, 8E0W7CZ, 8E0W8CZ)
Metastatic cancer	C77-C79
Anastomotic leak	K63.2, K65.1, K91.3, K91.81, K91.89
Arrhythmia	I44.0, I44.1, I44.2, I44.30, I45.89, I48.91, I48.92
Infection	L00-L08, A00-B99, T81.43, T81.49, O86.03, Z16
Sepsis	R78.81, A41, R65.2, A42.7, A22.7, B37.7, A26.7, A28.2, A54.86, A32.7, A39.2, A20.7, A21.7, A48.3, A24.1
Pneumonia	A48.1, J12 - J18
Respiratory failure	J95.2-J95.8, J96.00, J96.90, J80, J81.0
Mechanical ventilation	Z99.12, ICD-10-PCS: 5A1935Z, 5A1945Z, 5A1955Z
Dysphagia	R13.1, R47.02, D50.1
VTE	I26.0, I26.9, I80.0 -I80.3, I80.8, I80.9, I81, I82, O08.2, O22.3, O87.1, O88.2
CVA	I60, I61, I63, I69
AMI	I21
Bleeding	E36.0, G97.32, H59.11, I97.41, I97.12, J95.61, J95.62, K91.61, K91.62, L76.01, D78.21, D78.22, E89.81, G97.3, G97.5, H59.11, H59.12, H59.31, H59.32, H95.2, H95.4, I97.6, J95.83, K91.6, K91.84, L76.0, L76.2, M96.81, M96.83, N99.6, N99.82
Shock	R57, T81.1, T88.2, R65.21, A48.3
Urinary Tract Infection	N39.0
Acute kidney injury	N17
Smoking	Z71.6, Z72.0, Z86.43, Z87.891, F17, O99.33, T65.2
Congestive heart failure	I09.9, I11.0, I13.0, I13.2, I25.5, I42.0, I42.5-I42.9, I43, I50, P29.0
Hypertension	I10-I16, I1A
Hypothyroidism	E03
Diabetes	E10-E13
Obesity	E66, Z68.3, Z68.4

Chronic pulmonary disease	I27.8, I27.9, J40 -J47, J4A, J68.4, J70.1, J70.3
Chronic kidney disease	I12.0, I13.1, N03.2-N03.7, N05.2-N05.7, N18, N19, N25.0, Z49.0-Z49.2, Z94.0, Z99.2
Severe Liver disease	I85.0, I86.4, K70.4, K71.1, K72.1, K72.9, K76.5, K76.6, K76.7
Rheumatic disease	M05, M06, M31.5, M32-M34, M35.1, M35.3, M36.0

MIE, minimally invasive esophagectomy; RAMIE, robot-assisted minimally invasive esophagectomy; VTE, venous thromboembolism; CVA, cerebral vascular accident; AMI, acute myocardial infarction.

Supplemental Table S2. Categories of hospital bed numbers

Hospital bed numbers category (beginning from 1998) *			
Location and teaching status	**Small**	**Medium**	**Large**
NORTHEAST			
Rural	1-49	50-99	100+
Urban, nonteaching	1-124	125-199	200+
Urban, teaching	1-249	250-424	425+
MIDWEST			
Rural	1-29	30-49	50+
Urban, nonteaching	1-74	75-174	175+
Urban, teaching	1-249	250-374	375+
SOUTHERN			
Rural	1-39	40-74	75+
Urban, nonteaching	1-99	100-199	200+
Urban, teaching	1-249	250-449	450+
WESTERN			
Rural	1-24	25-44	45+
Urban, nonteaching	1-99	100-174	175+
Urban, teaching	1-199	200-324	325+

* The definition of hospital bed numbers in the Healthcare Cost and Utilization Project (HCUP) may vary based on the year, geographic region, and hospital classification. (https://hcup-us.ahrq.gov/db/vars/hosp_bedsize/nisnote.jsp)

Supplemental Table S3. Characteristics of the study population before propensity score matching.

Characteristics	Total (n= 1,198)	Surgical types RAMIE (n= 314)	MIE (n= 884)	p-value
**Outcomes**				
**In-hospital mortality**	17 (1.4)	6 (1.9)	11 (1.2)	0.328
**Unfavorable discharge** [a]	81 (6.9)	17 (5.5)	64 (7.3)	0.238
**Prolonged LOS** [a, b]	245 (20.7)	64 (20.8)	181 (20.7)	0.986
**Total hospital costs**	167.5 ± 6.5	187.9 ± 7.7	160.2 ± 7.8	**0.019**
**Complications**	490 (40.9)	132 (42.0)	358 (40.5)	0.617
Infection	119 (9.9)	27 (8.6)	92 (10.4)	0.307
Sepsis	49 (4.1)	12 (3.8)	37 (4.2)	0.754
Pneumonia	55 (4.6)	14 (4.5)	41 (4.6)	0.889
Respiratory failure	107 (8.9)	33 (10.5)	74 (8.4)	0.226
Mechanical ventilation	72 (6.0)	25 (8.0)	47 (5.3)	0.063
Dysphagia	239 (19.9)	70 (22.3)	169 (19.1)	0.215
VTE	36 (3.0)	10 (3.2)	26 (2.9)	0.815
CVA	7 (0.6)	2 (0.6)	5 (0.6)	0.870
AMI	9 (0.8)	0 (0.0)	9 (1.0)	-
Bleeding	8 (0.7)	0 (0.0)	8 (0.9)	-
Shock	36 (3.0)	10 (3.2)	26 (2.9)	0.792
Urinary Tract Infection	31 (2.6)	5 (1.6)	26 (2.9)	0.186
Acute kidney injury	82 (6.8)	21 (6.7)	61 (6.9)	0.895
**Demography**				
Age, years	63.4 ± 0.4	61.5 ± 0.6	64.0 ± 0.4	**0.004**
20-29	16 (1.3)	6 (1.9)	10 (1.1)	**0.009**
30-39	41 (3.4)	13 (4.1)	28 (3.2)	
40-49	110 (9.2)	33 (10.5)	77 (8.7)	
50-59	240 (20.0)	72 (22.9)	168 (19.0)	
60-69	385 (32.1)	89 (28.3)	296 (33.5)	
70-79	305 (25.5)	87 (27.7)	218 (24.7)	
80+	101 (8.4)	14 (4.5)	87 (9.8)	
Sex				0.053
Male	751 (62.7)	211 (67.2)	540 (61.1)	

Female	447 (37.3)	103 (32.8)	344 (38.9)	
Insurance status / Primary Payer				0.540
Medicare/Medicaid	668 (55.9)	170 (54.5)	498 (56.4)	
Private including HMO	469 (39.2)	129 (41.3)	340 (38.5)	
Self-pay/no-charge/ other	58 (4.9)	13 (4.2)	45 (5.1)	
Missing	3	2	1	
**Smoking**	578 (48.2)	164 (52.2)	414 (46.8)	0.094
**Major comorbidities**				
Congestive heart failure	77 (6.4)	17 (5.4)	60 (6.8)	0.360
Hypertension	708 (59.1)	186 (59.2)	522 (59.0)	0.951
Hypothyroidism	120 (10.0)	23 (7.3)	97 (11.0)	**0.040**
Diabetes	251 (21.0)	66 (21.0)	185 (20.9)	0.971
Obesity	267 (22.3)	70 (22.3)	197 (22.3)	0.998
Chronic pulmonary disease	255 (21.3)	60 (19.1)	195 (22.1)	0.230
Chronic kidney disease	85 (7.1)	25 (8.0)	60 (6.8)	0.494
Severe Liver disease	6 (0.5)	1 (0.3)	5 (0.6)	0.584
Rheumatic disease	35 (2.9)	9 (2.9)	26 (2.9)	0.943
**Weekend admission**	16 (1.3)	2 (0.6)	14 (1.6)	0.089
**Hospital bed numbers**				**<0.001**
Small	113 (9.4)	43 (13.7)	70 (7.9)	
Medium	203 (16.9)	36 (11.5)	167 (18.9)	
Large	882 (73.6)	235 (74.8)	647 (73.2)	
**Location/teaching status**				0.214
Rural	18 (1.5)	7 (2.2)	11 (1.2)	
urban nonteaching	90 (7.5)	21 (6.7)	69 (7.8)	
urban teaching	1090 (91.0)	286 (91.1)	804 (91.0)	

RAMIE, robot-assisted minimally invasive esophagectomy; MIE, conventional minimally invasive esophagectomy; VTE, venous thromboembolism; CVA, cerebral vascular accident; AMI, acute myocardial infarction.

Continuous variables are presented as mean ± SE.

Categorical variables are presented as unweighted counts (weighted percentage).

P-value < 0.05 are shown in bold.

[a] Excluded patients with in-hospital mortality.

[b] Length of stay > 75th percentile (10 days).
